# The biophysics of disordered proteins from the point of view of single-molecule fluorescence spectroscopy

**DOI:** 10.1042/EBC20220065

**Published:** 2022-12-16

**Authors:** Jasmine Cubuk, Melissa D. Stuchell-Brereton, Andrea Soranno

**Affiliations:** 1 Department of Biochemistry and Molecular Biophysics, Washington University in St Louis, 660 St Euclid Ave, Saint Louis, MO 63110, U.S.A.; 2 Center for Science and Engineering of Living Systems, Washington University in St Louis, 1 Brookings Drive, Saint Louis, MO 63130, U.S.A.

**Keywords:** Fluorescence Correlation Spectroscopy, Forster Resonance Energy Transfer, intrinsically disordered proteins, single molecule fluorescence spectroscopy

## Abstract

Intrinsically disordered proteins (IDPs) and regions (IDRs) have emerged as key players across many biological functions and diseases. Differently from structured proteins, disordered proteins lack stable structure and are particularly sensitive to changes in the surrounding environment. Investigation of disordered ensembles requires new approaches and concepts for quantifying conformations, dynamics, and interactions. Here, we provide a short description of the fundamental biophysical properties of disordered proteins as understood through the lens of single-molecule fluorescence observations. Single-molecule Förster resonance energy transfer (FRET) and fluorescence correlation spectroscopy (FCS) provides an extensive and versatile toolbox for quantifying the characteristics of conformational distributions and the dynamics of disordered proteins across many different solution conditions, both *in vitro* and in living cells.

## Introduction

The last 20 years have seen the emergence of a class of proteins that challenge the classic structure–function paradigm [1]. Indeed, a large fraction of proteins in the eukaryotic proteome are completely or partially unstructured [2], but nevertheless play essential roles in biological function [3–6], ranging from transcription [7–10] and translation [11–13] to transport processes [14] and membrane organization [15–17]. Their ‘shape shifting’ nature upon binding [18] and the occurrence of short linear-interacting motifs [19,20] make them essential components of cellular signaling, allowing for specificity with a multiplicity of signaling targets [19–23]. The multivalence of interactions encoded in their sequence can also play an essential role in regulating self-assembly processes [3,24]. Because of these key roles, disordered proteins are also central to several diseases, such as neurodegeneration and cancer [25,26].

**Figure 1:**
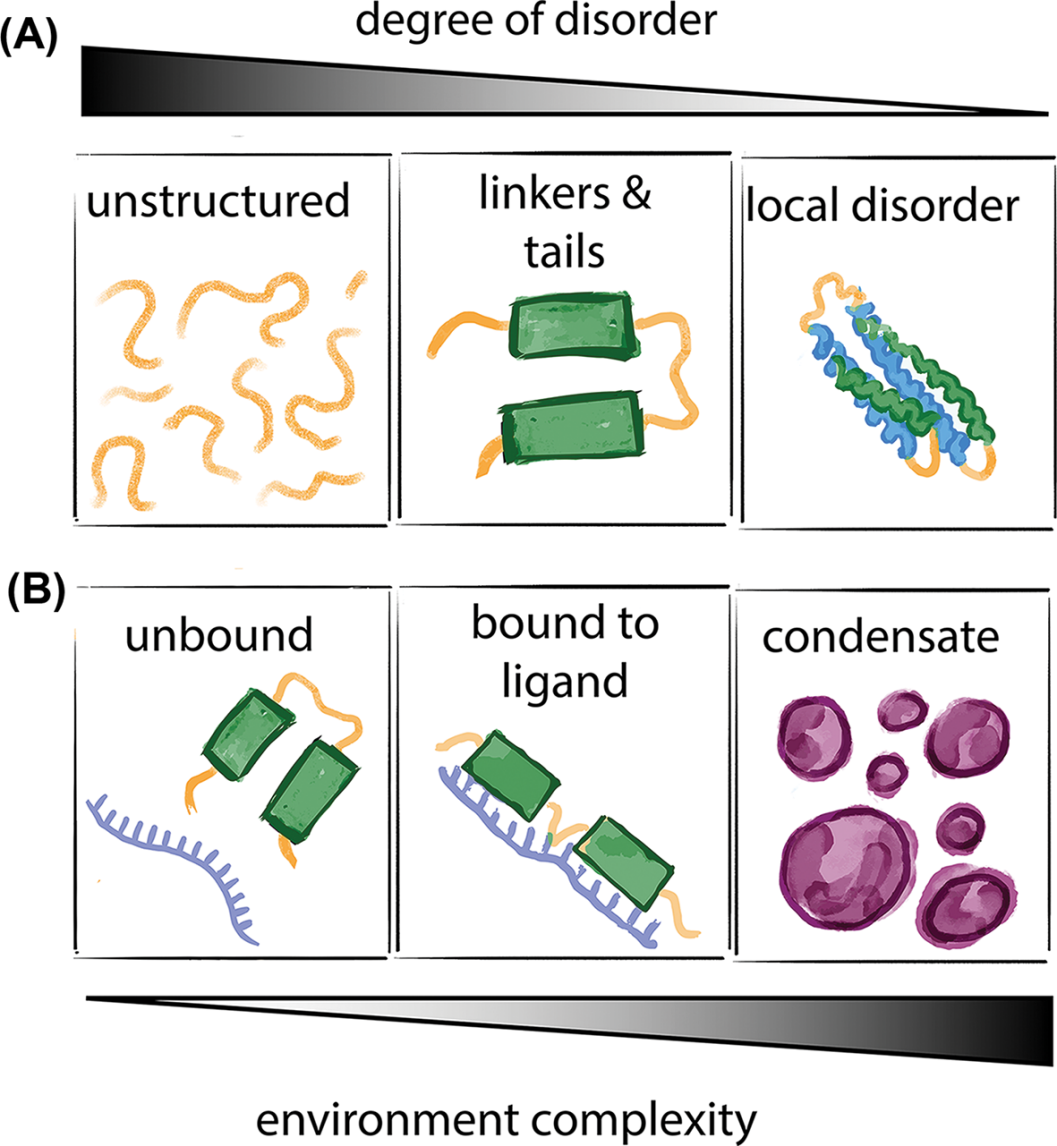
Spectrum of conformational disorder A whole spectrum of disorder can occur in proteins, from completely unstructured sequences to disordered linkers and tails flanking folded domains or just short loops that introduce local disorder in mostly folded proteins. In addition, given the lack of a stable 3D structure, the complexity of the solution (*in vitro* and in the cell) can modulate conformations and dynamics of the disordered protein, whether the protein is studied in isolation, bound to a ligand, within a biomolecular condensate or other crowded environment.

There is a whole spectrum of conformational heterogeneity in proteins, where disorder can span either the whole protein, some regions of the protein (e.g. tails and linkers that flank and connect folded domains), or just short loops that are involved in the organization of structured proteins. The environment ‘complexity’ can further modulate the properties of disordered proteins, since conformations and dynamics will depend on whether proteins are studied in isolation, in presence of ligands, or within the crowded milieu of a cell (Figure 1).

These intrinsically disordered proteins (IDPs) and regions (IDRs) do not adopt a unique stable structure, but instead sample many different conformations. As a result, their flexible nature cannot be interpreted in the terms of classical structural biology and requires a different language to describe the properties of these conformational ensembles. At the same time, different experimental approaches are required to access and quantify protein conformations, dynamics, and interactions.

The physics of polymers has emerged as a powerful framework to identify key observables and explain the thermodynamic-driving forces controlling IDPs [27–30]. In parallel, single-molecule fluorescence spectroscopy has provided means to directly test and quantify predictions of polymer models. In this review, we have focused on recounting the major findings of the biophysics of disordered proteins in a simple and accessible form, discussing how single-molecule fluorescence spectroscopy can be harnessed to access such properties. We hope this brief summary can provide an entry point in the investigation of IDPs biophysics with single-molecule tools.

## Single-molecule fluorescence

**Figure 2:**
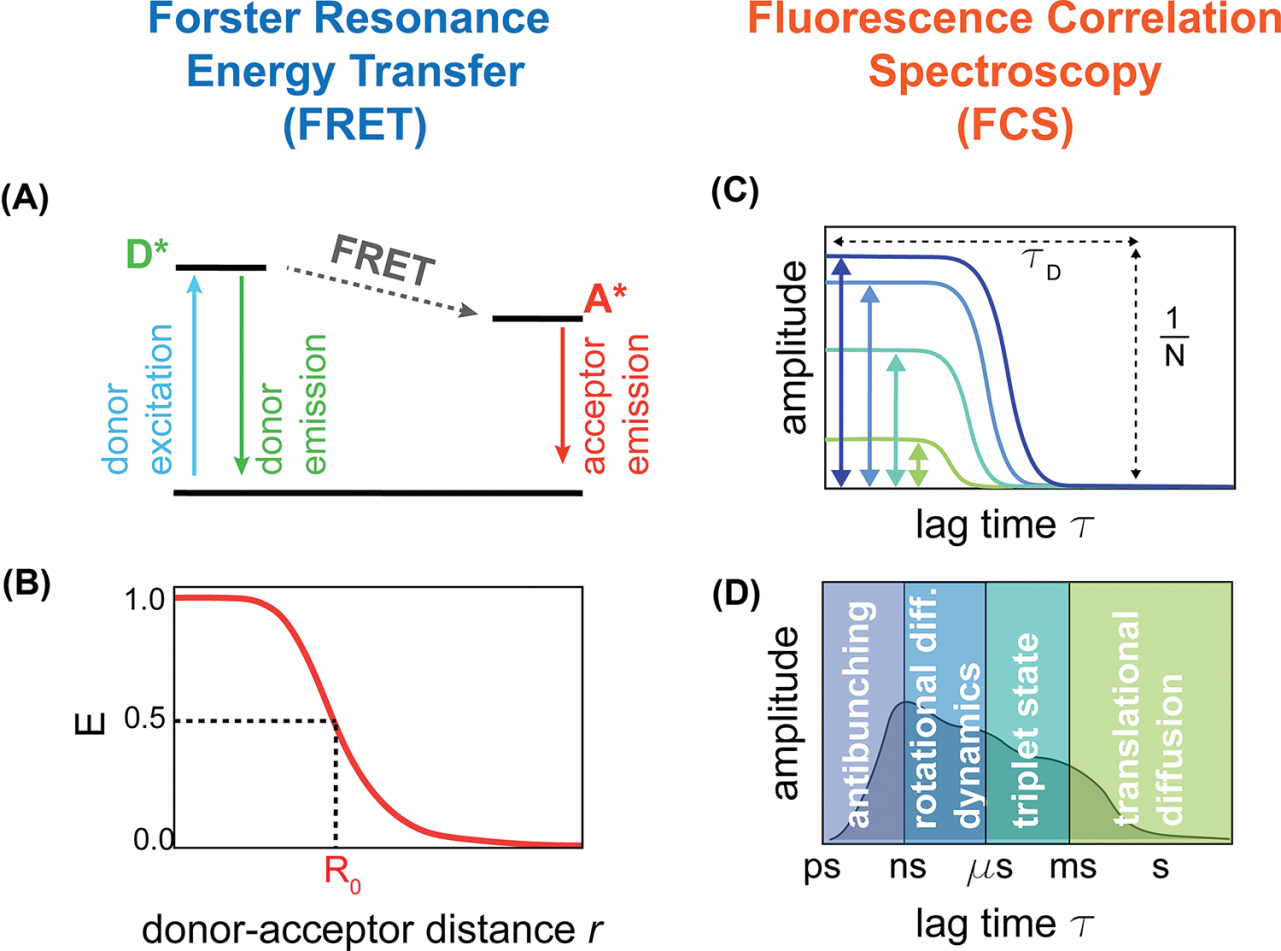
Single-molecule fluorescence spectroscopy (**A**) In Förster resonance energy transfer (FRET), donor excitation can either result in a photon emission from the donor itself or a nonradiative energy transfer to the acceptor with consequent emission of an acceptor photon without a direct excitation of the acceptor. (**B**) The efficiency of the energy transfer process depends on the interdye distance *r* to the power of 6 and on the characteristic Förster radius, which corresponds to the distance at which the efficiency is equal to 50% (compare with eqn 1). (**C**) In fluorescence correlation spectroscopy (FCS), the amplitude of the correlation reports about the inverse of the number of molecules *N* in the observation volume, whereas the shift in decay represents the characteristic time of decorrelation. In typical confocal experiments of freely diffusing molecules, one of the sources of decorrelation is the diffusion of the molecule in and out of the confocal volume and the characteristic time is the diffusion time *τ*_D_ (compare with eqns 5 and 6). (**D**) Other timescales that can be studied in FCS experiments are related to the excitation of the fluorophore (antibunching), the rotational diffusion of the molecule and intrachain dynamics, and the photophysics of the triplet state.

**Figure 3:**
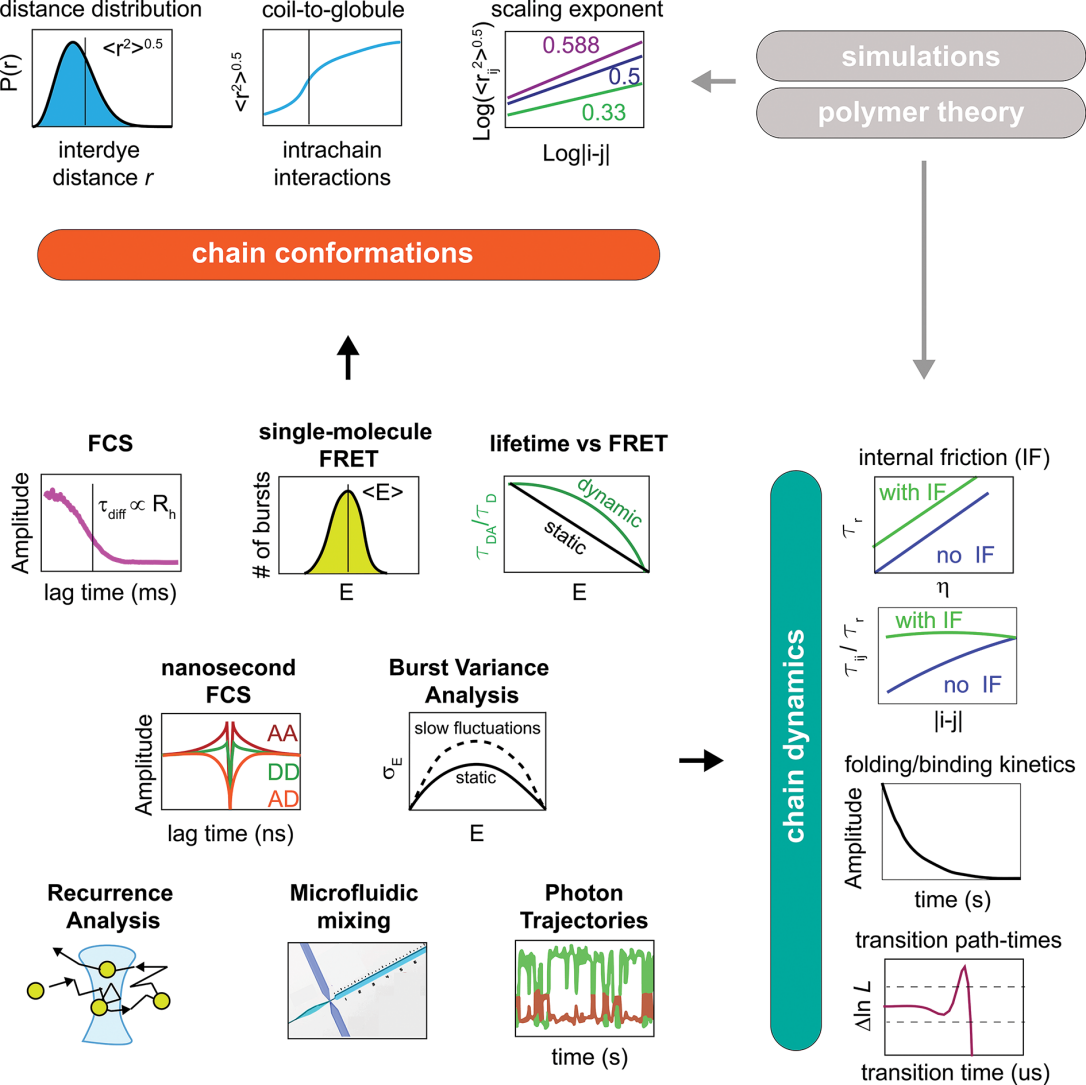
Experimental methods for the study of disordered proteins Single-molecule fluorescence offers a versatile set of tools for the investigation of conformations and dynamics in IDPs and IDRs. FCS provides access to the diffusion time *τ*_diff_ and corresponding hydrodynamic radius *R*_H_ (see eqn 6). Single-molecule FRET provides access to the mean transfer efficiency, whereas the fluorescence lifetime provides constraints for estimating the shape of the distribution. The root-mean-square interdye distance or *R*_H_ can be used to study the coil-to-globule transition while modulating interactions (e.g. denaturant or temperature). Dependence of the interdye distance with the sequence separation between the dyes provides access to the scaling exponent. Nanosecond-FCS provides access to fast dynamics, from nanoseconds to milliseconds and enables disentangling the contribution of solvent and internal friction, either by explicitly studying the viscosity of the solution or by monitoring dynamics as function of sequence separation. Burst variance analysis (BVA) provides an alternative method to quantify slow dynamics in the microsecond to millisecond timescale. Photon trajectories provide access to kinetics of folding and binding of IDPs and transition path-times. Recurrence analysis and microfluidics enable further investigation of kinetics associated with conformational changes in different solvents or upon binding ligands. The experimental quantities can be further compared with simulations or polymer models.

Single-molecule fluorescence spectroscopy offers a versatile toolbox to investigate the conformations and dynamics of disordered proteins across many solution conditions (Figures 2 and 3). The major advantages of single-molecule methods compared with classical ensemble approaches are: (i) the possibility of resolving distinct conformational ensembles, allowing for distinguishing structural conformational changes occurring with solvent conditions or upon binding with ligands; (ii) the ability of accessing protein dynamics contextually with information on the conformational ensemble; (iii) the particularly low concentration regime (picomolar range), which enables access to the monomeric form of the protein, even for aggregation and oligomerization prone sequences.

The two major single-molecule fluorescence approaches that are commonly applied to IDPs rely on FRET and FCS.

FRET provides a molecular spectroscopic ruler to measure distances on the length scale of nanometers [31]. FRET experiments involve the attachment of two fluorophores to the protein of interest and require that the emission spectra of one fluorophore (the donor) overlaps with the absorption spectrum of the other fluorophore (the acceptor). When this condition is satisfied, the excitation of the donor fluorophore can lead to the direct emission of a donor photon or to a nonradiative energy transfer toward the acceptor and the consequent emission of an acceptor photon (Figure 2A). The efficiency of this energy transfer is given by (eqn 1):

(1) $$E(r)=\frac{R_{0}^{6}}{R_{0}^{6}+r^{6}}$$

where *r* is the distance between the donor and the acceptor fluorophore and *R*_0_ is the Förster radius. The Förster radius sets the distance at which the transfer efficiency is equal to 50% and depends on the spectroscopic properties of the dyes, their reciprocal orientation, and the refractive index of the solution (Figure 2B).

Single-molecule detection is achieved by limiting the number of molecules in the volume of observation. This can be directly realized by sparsely immobilizing molecules on the surface, such that the molecules are statistically sufficiently separated from each other, or by studying freely diffusing molecules and diluting the sample such that statistically only one molecule is observed at a time. Typical experiments make use of either total internal reflection fluorescence (TIRF) or confocal microscope setups.

In TIRF experiments, the total internal reflection enables a thin layer of the solution (<100 nm from the coverslip) to be illuminated by an evanescent wave and the detection of excited fluorescent molecules is performed via a camera-based setup. The illumination of a small layer of solution allows studying interactions in presence of a high concentration of fluorescence proteins (e.g. ligands), since only the molecules close to the surface will be excited; however, it requires close proximity (normally, immobilization) of the sample to the surface. The camera-based setup enables the simultaneous detection of multiple molecules, though time resolution of fast events is limited by the camera frame rate (commonly in the tens of milliseconds timescale).

In single-molecule confocal setups, a laser beam is coupled into a microscope objective with a high numerical aperture, which focuses the beam into a diffraction-limited spot within the sample. Emitted photons are detected through the same objective, filtered through a small pinhole (between 30 and 150 nm), and finally separated and detected on single-photon avalanche photodiodes [32]. This type of setup is often coupled with fast electronics for single photon detection, allowing for molecules and fluorophore photophysics to be studied over a broad range of timescales, from picoseconds to minutes [31]. Confocal microscopy allows for both studying freely diffusing and immobilized molecules. However, for immobilized molecules, it requires focusing on each single molecule individually, making the acquisition time for multiple immobilized molecules significantly longer compared with the one obtained by TIRF microscopy; on the positive side, this approach commonly allows for much higher time resolution on the fast timescales, enabling access to fluorescence lifetimes and anisotropy.

In both TIRF and confocal single-molecule FRET, the transfer efficiency *E* is quantified by counting the number of donor and acceptor photons, *n*_D_ and *n*_A_, according to (eqn 2):

(2) $$E=\frac{n_{A}}{\left(n_{A}+n_{D}\right)}$$

If fluorescence lifetime is accessible, transfer efficiency can be also evaluated by comparing the lifetime of the donor in presence and in absence of the acceptor (eqn 3):

(3) $$E=\frac{1-\tau_{DA}}{\tau_{D}}$$

where *τ*_D_ is the fluorescence lifetime of the donor and *τ*_DA_ is the fluorescence lifetime of the donor in the presence of the acceptor.

FCS provides an alternative set of approaches to investigate protein conformations (the overall size) and dynamics. The concept of fluorescence correlation was originally introduced in the 1970s by Elson, Magde, and Webb [33–35] and relies on studying fluctuations in the intensity of fluorescence signals as a reporter for protein diffusion and concentration. This can be understood easily by considering a sufficiently dilute solution, where the passing of fluorescent molecules through the detection volume leads to significant changes in the detected intensity compared with the average background. This can be quantified by studying the correlation of the fluorescence fluctuations [36,37] (eqn 4):

(4) $$G(\tau )=\frac{<F(t)F(t+\tau )>}{<F(t){>}^{2}}$$

where *F*(*t*) represents the fluorescence intensity at the time *t*, <> represents the temporal average over all times *t*, τ is the lag time at which the correlation *G*(τ) is computed. Under the assumption of a Gaussian 3D profile, the correlation can directly be linked to the diffusion time and concentration of molecules (eqn 5):

(5) $$G_{Diff}(\tau )=\frac{1}{N}{\left(1+\frac{\tau }{\tau_{Diff}}\right)}^{-1}{\left(1+\frac{\tau }{\tau_{Diff}}.\frac{r_{0}^{2}}{z_{0}^{2}}\right)}^{-0.5}$$

where *N* is equivalent to the average number of molecules in the detection volume, *τ*_Diff_ is the average time of molecules diffusing through the detection volume and *r*_0_ and *z*_0_ are the lateral and axial radial distances of the confocal volume, respectively (Figure 2C). While the amplitude of the correlation directly reports on the average number of molecules in the detection volume, *τ*_Diff_ reports about the diffusion coefficient of the molecule and can be related to its hydrodynamic Stokes-radius, *R*_H_ (eqn 6):

(6) $$R_{H}=\frac{2K_{B}T\;\tau_{Diff}}{3\pi \eta r_{0}^{2}}$$

where *K*_B_ is the Boltzman constant, *T* is the absolute temperature, and *η* is the viscosity of the solution at a given *T*.

FCS measurements are commonly performed in a confocal setup and therefore are compatible with single-molecule FRET experiments. Indeed, FCS is not only limited to measure diffusion and concentration of molecules: depending on the source causing fluctuations in fluorescence intensity, FCS can provide access to static quenching and other photophysical properties of the dyes as well as dynamics within the molecule of interest (Figure 2D).

## Accessing the disordered state

In 1999, the labs of DeGrado and Hochstrasser proved the feasibility of single-molecule FRET experiments on proteins, investigating the denaturation of immobilized [38] and freely diffusing [39] GCN-4 fragments. Contextually, Deniz et al. [40] demonstrated how this approach enables distinguishing and separating properties associated with the folded and unfolded states. Specifically, their measurements of the chymotrypsin inhibitor 2 identified changes of the unfolded state as a function of the denaturant, which the authors speculated could be due to loss of secondary structure or increase in solubility of the disordered state. Schuler et al. [41] observed analogous changes for the Cold shock protein of *Thermotoga Maritima* and, using polyproline sequences as control measurements, ruled out that the observations were due to photophysical effects, and indeed represented changes in the conformations of the unfolded state. These works set the stage for investigating the properties of the denatured state of foldable proteins, which represents another occurrence of ‘disorder’ in proteins.

In the case of denatured proteins, the mean transfer efficiency associated with the distribution of the unfolded state represents a mean value of the transfer efficiencies associated with the different configurations explored by the chain: because chain dynamics are significantly faster (nanosecond timescale) than the burst detection (millisecond) in single-molecule experiments, the different transfer efficiencies are averaged out [31]. The same applies to disordered proteins. The distance distribution associated with the denatured or disordered protein is often sufficiently well-described by simple polymer models (such as the Gaussian chain [42], Worm-like chain [43], and Self-Avoiding Walk distributions [29,44,45]), or can be estimated from simulations [28,46–48].

The work of Sherman and Haran [49] on the unfolded state of protein L introduced an important explanation for the conformational change of the disordered state when increasing denaturant, linking the observed phenomenon to the coil-to-globule transition in polymers [49–51]. Indeed, depending on the balance of the monomer–monomer and monomer–solvent interactions, polymers can adopt conformations of a compact globule (when monomer–monomer interactions dominate) or expanded conformations (when solvent–monomer interactions are favored) [42]. Varying the strength of monomer and solvent interactions leads to a transition between these two extreme cases and results in the so-called coil-to-globule transition [52]. Examples of interactions include excluded volume interactions (the physical occupancy of space of the residues), electrostatic interactions, and hydrophobic interactions. A particular case occurs when attractive and repulsive interactions in the system (protein and solvent) cancel each other: this particular condition is referred to as the ‘theta state’ or ‘theta solvent condition’ and is a key reference state in polymer physics. Altogether, the coil-to-globule framework provides a physical explanation for the change in conformations of the unfolded (either denatured or intrinsically disordered) state in presence of denaturant, where the denaturant acts as a better solvent and favors more expanded conformations.

**Figure 4:**
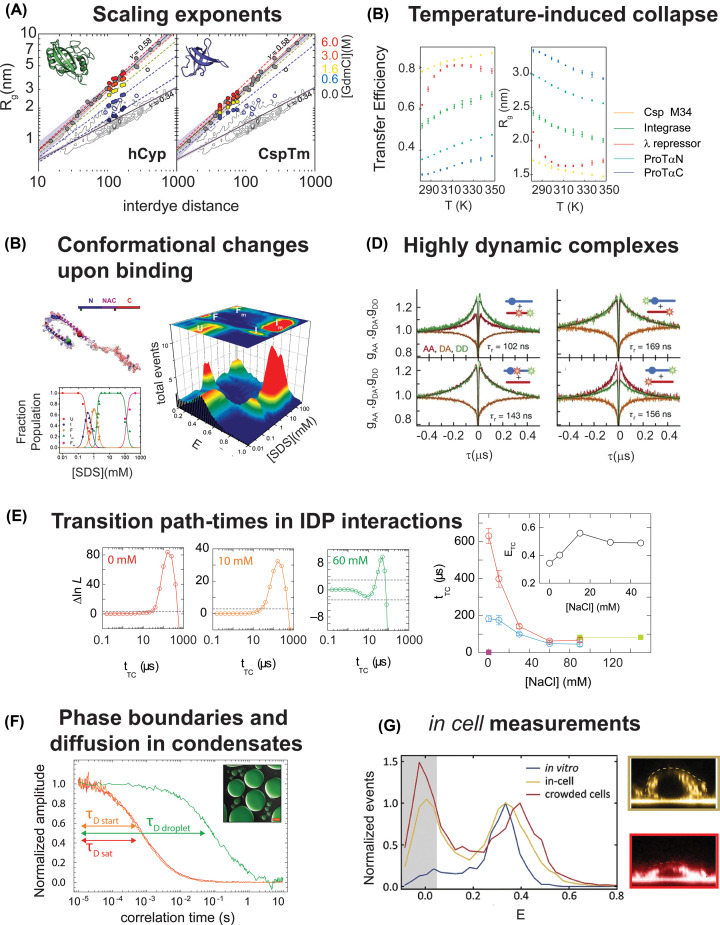
Examples of single-molecule fluorescence measurements (**A**) Measurements of the interdye distance (represented in terms of apparent radius of gyration) as function of interdye sequence separation for the unfolded state of two proteins, human CyclophilinA (hCyp) and Cold-shock protein from *Thermatoga maritima* (CspTm), reveal changes in the scaling exponent from low to high values with increasing denaturant concentration (adapted from ref. [53]). (**B**) Temperature induced collapse of five different disordered regions, including a destabilized mutant of Cold-shock protein, the N-terminal disordered tail of HIV-Integrase, λ repressor, and the N- and C-terminal halves of Prothymosin alpha (adapted from ref. [93]). (**C**) Conformational changes of alpha synuclein upon binding with SDS vesicles. Single-molecule FRET enables resolving different intermediates and bound states as a function of SDS concentration (adapted from ref. [122]). (**D**) Nanosecond FRET-FCS reveals fast dynamics in the complex between Prothymosin alpha and histone H1. Autocorrelation of donor–donor (DD) and acceptor–acceptor (AA) exhibits a correlated decay, whereas the cross-correlation (AD) shows an anticorrelated increase in the signal. A relaxation time is extracted by globally fitting the three correlations. (compare with eqn 7). The rapid increase in all the correlations at a few nanoseconds is related to the excitation rate of the fluorophore and reflects the fact that a single fluorophore can emit only a photon at a time: this is commonly referred to as antibunching. (**E**) Confocal measurements of tethered molecules provide access to kinetics in the interaction of TAD and NCBD, including quantification of the time spent in short-lived states such as the transition path-time, as a function of NaCl concentration (adapted from ref. [127]). (**F**) FCS measurements provide quantification of concentrations inside and outside of phase separated solutions as well as access to the diffusion time of the fluorescent molecules in both phases (adapted from ref. [138]). Concentrations of labeled components can be estimated from the amplitude of the correlation, whereas diffusion time is measured from the decay time of the correlation (compare eqn 5). (**G**) *In cell*, single-molecule FRET measurements of Prothymosin alpha (yellow curve) reveal modulation of conformational changes compared with *in vitro* measurements (blue curve), which are amplified by inducing osmotic stress and increasing the concentration of molecules in the cell (red curve). Adapted from ref. [137].

This hypothesis has been further tested by Hofmann et al. [53]. Here, they harnessed the ability of measuring interdye distances within a protein by attaching fluorophores at different positions. This approach allows directly estimating the scaling exponent [54] of the disordered state under different solvent conditions, for both folded and IDPs. The scaling exponent relates the root-mean-square interdye distance <*r*^2^>^0.5^ to the degree of polymerization of the molecule (in this case, the number of peptide bonds in the protein) and is directly related to the three fundamental states identified in the coil-to-globule transition. <*r*^2^>^0.5^ is proportional to *N*^1/3^ for the case of a globule, to *N*^1/2^ for the case of an ideal chain, and to *N*^0.588^ for the case of a chain dominated by excluded volume effects. Hofmann et al. [53] found that in high denaturant different sequences follow the scaling exponent expected for a chain in a good solvent, whereas under native conditions different scaling exponents are observed depending on the sequence properties (charge, hydrophobicity, etc.) (Figure 4A). This observation was consistent with predictions from simulations [55,56] and with previous estimates of the scaling exponent of folded proteins in denaturant [57] and has been further corroborated in subsequent works exploring the role of denaturant on unfolded proteins [58–60]. FRET measurements have been also compared with FCS measurements and dynamic light scattering to confirm the expansion of the disordered state of proteins with increasing denaturant concentration [49,53,61,62].

The information from single-molecule FRET is often integrated with that provided by other approaches, which can help in defining the properties of the disordered ensemble. This includes measurements of the radius of gyration via SAXS [45,59,61,63–67], local conformations via NMR [65–68], and simulations [28,46–48,63,65,67,69].

## Effect of sequence on chain conformations

The large spectrum of scaling exponents observed under native conditions for different unfolded and disordered proteins [53,64,70,71] reflects the contribution of sequence composition on the conformations of disordered ensembles. Müller-Späth et al. [72] used single-molecule FRET to probe the conformations of unfolded proteins, showing the degree of compaction and expansion of the interdye distance depends on the charged residue content in the protein, in good agreement with prediction from polymer theories [73–75]. Indeed, the theory of polyelectrolytes (polymers whose monomers carry all the same type of charge) and polyampholytes (polymers carrying both positive and negative charges) indicate that the dimension of charged polymers depends on the contribution of the total net charge of the protein (which favors the expansion of the chain) and the total number of charged residues of the chain (which represents local compaction along the chain due to the positive and negative residues in the chain). These observations matched independent computational work from the Pappu group [76], which further demonstrated how the net charge, total number of charged residues, and charge patterning can modulate the conformations of IDRs, favoring extended, compact, and more exotic conformations such as tadpoles and hairpins [77]. Advanced polymer models have been further developed to capture sequence-specific effects of charge distribution [78–80], revealing how the pattern of charges in polyampholyte sequences can even lead to coexistence of two distinct ensembles of conformations [81,82]. Importantly, parameters such as net charge and total charge are influenced by the ionization of charged groups, which may be affected by the local density of charged residues [83]. Polyampholyte and polyelectrolyte theories capture also the conformational response to salt, including effects of charge screening and counterion condensation [73,75,80,84,85].

While charged residues are an important determinant of IDPs/IDRs conformations, other amino acids can play a fundamental role on protein conformations [29]. For example, a comparative analysis of the hydrodynamic radii of different IDPs identified the content in proline residues as an essential factor modulating the disordered conformational ensemble [86]. SAXS measurements and simulations of Ash1 further revealed that proline residues can favor expanded configurations even when the charge content of the protein is changed by phosphorylation and more compacted conformations are expected to be populated [87]. Understanding the effect of sequence composition is essential to rationalize the conformational response to solution conditions and how this impacts the interaction with ligands. This is particularly important in the case of linkers, where sequence composition can modulate the local effective concentration of interacting regions [88,89].

## Effect of temperature

Temperature modulation provides a wealth of information regarding the entropic and enthalpic contributions controlling molecular conformations and interactions [90]. This is often realized using custom built temperature-controlled chambers, where the sample temperature can be directly controlled and calibrated against a well-known standard (e.g. the temperature-dependent lifetime of rhodamine B) [90–92].

Single-molecule FRET experiments on IDRs and unfolded proteins as a function of temperature [91,93] have revealed a surprising result for the temperature dependence of chain dimensions. With increasing temperature, the disordered state conformations first undergo compaction before starting to expand again (Figure 4B). Similar results have been reported also for different IDPs by dynamic light scattering [94] and small angle X-ray [95,96] measurements. At first glance, these observations appear counterintuitive. Based on the classical dependence of the coil-to-globule observed in polymers [52], one would expect that a temperature increase would result in an expansion of the chain due to decrease in the strength of the interactions at play. The strengthening of interactions with increasing temperature points to a contribution of the ‘hydrophobic effect,’ but the extent of collapse is found to be amplified for hydrophilic charged sequences, not hydrophobic ones [93]. These observations can be rationalized by accounting for the temperature-dependent solvation free energies of the sequence amino acids [93]. The same temperature-induced collapse occurs when studying the unfolded state upon hot and cold denaturation [97,98], showing that the cold-denatured and hot-denatured state of a protein represents the same denatured state and their response to temperature follows the temperature trend of solvation-free energies [98].

## Accessing chain dynamics

While FRET is often used to determine conformational changes, it can also be used to investigate chain dynamics. Fast dynamics can be accessed by studying the autocorrelation of the donor and acceptor photons or the cross-correlation of donor and acceptor [99,100]. Use of zero-mode waveguides can shorten the acquisition time (down to tens of minutes) and enable measurements of very fast dynamics (low-nanoseconds) [101]. When studying fast dynamics with FCS, the correlation function in (eqn 5) (extends to (eqns 7a and 7b):

(7a) $$G(\tau )=\left(1-c_{ab}^{i,j}\frac{\tau }{\tau_{ab}^{i,j}}\right)\left(1+c_{CD}^{i,j}\frac{\tau }{\tau_{CD}}\right)\left(1+c_{T}^{i,j}\frac{\tau }{\tau_{T}^{i,j}}\right)G_{Diff}(\tau ),i=j$$

(7b) $$G(\tau )=\left(1-c_{ab}^{i,j}\frac{\tau }{\tau_{ab}^{i,j}}\right)\left(1-c_{CD}^{i,j}\frac{\tau }{\tau_{CD}}\right)\left(1+c_{T}^{i,j}\frac{\tau }{\tau_{T}^{i,j}}\right)G_{Diff}(\tau ),i\neq j$$

where *i* and *j* identify either the donor or acceptor photon emission, $c_{ab}^{i,j}$ and $\tau_{ab}^{i,j}$ represent the amplitude and relaxation time of the antibunching component, $c_{T}^{i,j}$ and $\tau_{T}^{i,j}$ capture the contribution in amplitude and relaxation time of the triplet state, and and τ*_CD_* reports about the amplitude and relaxation time of chain dynamics. Note that the relaxation timescale τ*_CD_* is the same across all correlations, whereas the sign associated with the chain dynamics amplitude $c_{CD}^{i,j}$ depends on whether this is is an autocorrelation (*i* = *j*) or a cross-correlation (i ≠ j). The reason for change of sign in the cross-correlation is intuitively understood by considering that FRET, as measured by comparing donor and acceptor emission, is inherently anticorrelated: an increase in the acceptor emission comes at the cost of a decrease in donor signal and vice versa (see Figure 4D).

This approach has enabled quantifying the timescale at which disordered proteins sample the distribution associated with their conformational ensemble, which is often in the order of tens or hundreds of nanoseconds. The measured quantity is the reconfiguration time, which is the time that it takes to the specific interdye distance to lose memory of the previous configuration. Chain dynamics are consistent with the expected behavior of simple polymer models [102–104], where the overall reconfiguration time of the chain is given by the sum of two contributions, one dependent on the solvent and therefore on viscosity, and one independent of the solvent and is usually referred to as internal friction. Internal friction may arise from different molecular sources, including dihedral angle rotation [105–107] and transient contact formation [108]. ns-FRET FCS provides an effective tool to quantify solvent and internal friction contributions. One method relies on studying the viscosity dependence of the reconfiguration time titrating a viscogen that slows down the dynamics of the chain: a linear extrapolation to zero viscosity provides a value that reports on internal friction contributions. Alternatively, chain dynamics across different segment lengths of the disordered region can provide access to the same quantities: in this case, internal friction causes deviations from the behavior of ideal chain, where the reconfiguration is expected to scale with the length of the segment [104]. Comparison with all-atom simulations [109], NMR [66,110], and Neutron Scattering [111,112] experiments supports this model and the importance of internal friction effects.

Photo-induced electron transfer (PET) FCS provides a complementary point of view [109,113–115]. Here, the autocorrelation decay reports about quenching of the fluorophore due to static contact formation with quenching residues (tryptophan, tyrosine, and histidines) or synthetic moieties. In this case, the rates of forming and breaking the static complex, *k_on_* and *k_off_*, define the amplitude *c_CD_* = *k_off_*/*k_on_* and the relaxation time τ*_CD_* = 1/(*k_off_*/*k_on_*) associated with chain dynamics (eqn 7a). Since the contact formation time measures the time required for two specific residues to come into contact, this time is usually longer than the reconfiguration time and the same polymer models mentioned above can be used to connect the two quantities. As expected based on the polymer models [103], contact formation is also impacted by internal friction [109].

While in the majority of cases, both FRET- and PET-FCS detect dynamics on the nanosecond time scale, this approach can indeed identify dynamics up to the diffusion time of the molecule (or longer, for immobilized molecules). Analysis of dynamics in the microsecond timescale can also be achieved by using BVA, which studies how the variance of transfer efficiency varies along the burst duration [116], or Hidden Markov analysis of the photon trajectory [117,118].

## Accessing ligand interactions

Single-molecule fluorescence spectroscopy can provide a direct readout for the interaction between disordered proteins and their ligands, whether these are small solutes (e.g. ions), another protein, or nucleic acids. Importantly, FRET enables the contextual study of conformations and dynamics, allowing discerning whether the interaction leads to folding upon binding [119–122] (Figure 4C), diffusion of the disordered protein on the folded-binding partner [123], or formation of a dynamic complex in which both components remain disordered (as in the case of prothymosin alpha and histone H1 [124,125] (Figure 4D)). Study of surface-tethered molecules enables quantification of the on- and off-rates of association via the analysis of the dwelling times in the bound and unbound state [120,126], as well as of transition path times [121,127] (Figure 4E). Alternatives for studying kinetics of interactions are offered by microfluidic mixing and recurrence analysis [128]. Microfluidic mixing provides common deadtimes of ∼1 ms, comparable with classic stop flow experiments [129–131]. Recurrence analysis relies on extremely dilute solutions, so dilute that there is a significant probability in short times to see the same molecule coming back (‘reoccurring’) in the confocal volume more than observing a new molecule [128]. This provides a direct tool to identify subpopulations in broad distribution histograms [123].

## IDPs in complex environments

The single-molecule fluorescence toolbox is constantly growing and advancements in the last decade have demonstrated the feasibility and relevance of investigating IDPs in a complex environment. Indeed, the lack of a stable 3D structure makes disordered proteins prone to changes due to the surrounding milieu. We have already discussed how salt, temperature, and interactions with other molecules can alter IDPs conformations and dynamics. This becomes even more relevant in the context of the intracellular medium. Indeed, the cell milieu is occupied by a significant fraction of components (proteins, nucleic acids, metabolites), which is sufficiently high to limit the available volume that each component can explore. This phenomenon is commonly referred to as crowding and impacts many aspects of proteins and nucleic acids, including folding, dynamics, interactions, and in the case of disordered proteins, also their conformations. Recent applications on both proteins [132,133] and nucleic acids [134,135] have now highlighted the advantages of single-molecule fluorescence spectroscopy in quantifying such contributions on a molecule of interest. Indeed, single-molecule FRET can provide a direct readout for the conformational and kinetic changes within crowded solutions [132,133] as well as probe the possibility of inducing ‘folded’ conformations in equilibrium with the disordered state [132]. One important finding is that disordered proteins sense crowding differently depending on their degree of expansion [133]: IDRs that are more expanded are more sensitive to changes in the crowded solution. This can be quantified and rationalized in terms of their scaling exponent. IDRs with a scaling exponent larger than 0.5 will collapse with increasing concentration of crowding agents, whereas IDRs with a scaling exponent equal or smaller than 0.5 will have less or negligible impact of the crowded milieu, as expected based on the theory of polymer mixtures [136]. A discussion of the polymer and colloidal theories that applies to crowding are reported in [71]. The trend observed *in vitro* with polymeric crowded solutions is also observed in cells, where the same proteins undergoes compaction when decreasing the available volume in the cell, e.g. by inducing osmotic stress [137]. The emerging role of membrane-less compartments in the cellular environment has pushed to investigate the driving forces controlling their assembly. In particular, IDRs have been largely investigated in the context of biomolecular condensates. Recent experiments have showcased the possibility of using: (i) FCS to determine concentrations and dynamics inside the dilute and dense phase of condensates [138–142]; (ii) single-molecule FRET to quantify the conformational changes of disordered proteins within the dense phase [143,144]. (Figure 4F,G)

## Summary

- Single-molecule fluorescence spectroscopy provides a versatile toolbox to investigate the biophysical properties of disordered proteins in isolation, when bound to another macromolecule, or within crowded environments.
- Measuring interdye distances via FRET enables studying contribution of the sequence composition and temperature effects on the dimensions of the disordered ensemble, providing direct access to physical parameters such as the scaling exponent.
- Nanosecond FRET- and PET-FCS provides access to fast chain dynamics and enables resolving solvent and internal friction contributions, whereas FCS captures conformational changes of the whole protein in terms of hydrodynamic radius.
- Integration of single-molecule fluorescence with orthogonal techniques such as SAXS, NMR, DLS, Neutron scattering, and simulations provides further constraints to capture the complexity of conformational ensembles along different length- and time-scales.
- Advancements in single-molecule spectroscopy and the growing understanding of the physical principles regulating disordered proteins pave the way to applying these methodologies to quantitatively understand the mechanisms of function and dysfunction.

## Abbreviations

BVA: burst variance analysis
DLS: dynamic light scattering
FCS: fluorescence correlation spectroscopy
FRET: Förster resonance energy transfer
IDP: intrinsically disordered protein
IDR: intrinsically disordered region
NMR: nuclear magnetic resonance
PET: photo-induced electron transfer
SAXS: small angle x-ray scattering
TIRF: total internal reflection fluorescence
